# Anodization of nanoporous alumina on impurity-induced hemisphere curved surface of aluminum at room temperature

**DOI:** 10.1186/1556-276X-6-596

**Published:** 2011-11-16

**Authors:** Chen-Kuei Chung, Ming-Wei Liao, Chun-Te Lee, Hao-Chin Chang

**Affiliations:** 1 Department of Mechanical Engineering, Center for Micro/Nano Science and Technology, and Advanced Optoelectronic Technology Center, National Cheng Kung University, Tainan, Taiwan 701, Republic of China

**Keywords:** anodic aluminum oxide, porous alumina, nanoporous template

## Abstract

Nanoporous alumina which was produced by a conventional direct current anodization [DCA] process at low temperatures has received much attention in various applications such as nanomaterial synthesis, sensors, and photonics. In this article, we employed a newly developed hybrid pulse anodization [HPA] method to fabricate the nanoporous alumina on a flat and curved surface of an aluminum [Al] foil at room temperature [RT]. We fabricate the nanopores to grow on a hemisphere curved surface and characterize their behavior along the normal vectors of the hemisphere curve. In a conventional DCA approach, the structures of branched nanopores were grown on a photolithography-and-etched low-curvature curved surface with large interpore distances. However, a high-curvature hemisphere curved surface can be obtained by the HPA technique. Such a curved surface by HPA is intrinsically induced by the high-resistivity impurities in the aluminum foil and leads to branching and bending of nanopore growth via the electric field mechanism rather than the interpore distance in conventional approaches. It is noted that by the HPA technique, the Joule heat during the RT process has been significantly suppressed globally on the material, and nanopores have been grown along the normal vectors of a hemisphere curve. The curvature is much larger than that in other literatures due to different fabrication methods. In theory, the number of nanopores on the hemisphere surface is two times of the conventional flat plane, which is potentially useful for photocatalyst or other applications.

**PACS: **81.05.Rm; 81.07.-b; 82.45.Cc.

## Background

Anodic aluminum oxide [AAO] can be classified into two types of structure, namely the barrier type and the porous type of structure. The barrier type with a thin and compact-packed structure has been widely used in protection and dielectric capacitors [1], while the porous-type structure has received much attention since the characteristic of a high-ordered nanopore arrangement was discovered [2]. Recently, many researches have been focused on the nanostructured materials due to some of their significant physical properties [3]. Although several techniques like photolithography, etching, or gas phase synthesis can produce nanowires or nanotubes [4], a template-assisted growing approach of nanoporous AAO is considered as one of the most prominent methods due to the advantages of a controllable diameter, high aspect ratio, and economical way in producing. The AAO template has been used in various applications such as multiple quantum wells [5], photonic crystals [6], light-emitting diodes [7], humidity sensors [8], nanomaterial syntheses [9], and supercapacitors [10]. Recently, a typical electrochemical method for producing AAO films was developed using a potentiostatic two-step anodization on costly high-purity (99.997%) Al films [2]. Other approaches such as pulse anodization [11] or isotropic etching [12,13] have been employed to fabricate three-dimensional nanostructures. Many conventional AAO templates were performed using direct current anodization [DCA] at a low temperature (0°C to 10°C) to avoid Joule-heat-dissolution effect at a relatively high room temperature. Also, AAO templates are preferred to grow on Al foil with a smooth surface in order to avoid the nonuniform electric field during the anodization process. Therefore, Al foil should be electropolished before anodization. However, porous alumina with forms of curved spheres has been reported in the anodization of Al films. Yin et al. [14] made nanopore patterns on a photolithography-and-wet-etched Al curved surface on a Si substrate by a conventional DCA method and discussed that the bending and branching features should be accounted for the interpore distance mechanism. In this article, we have synthesized AAO on a hemisphere curved surface using a hybrid pulse anodization [HPA] method [15,16] on low-purity (99%) Al foils at room temperature [RT]. HPA possesses more advantages than the conventional DCA not only in curbing the Joule heat, but also in operating at a higher RT due to effective cooling [15,17]. The high-curvature hemisphere surface was induced by the high-resistivity impurities in Al foil during the HPA process. It is noted that the defects and impurities in the low-purity Al foil can sometimes bring disturbing effects for locally enhancing oxidation and dissolution rates during the anodization process to produce a hemisphere curve on the Al foil surface after removal of the first-step AAO. The detailed growth behavior and mechanism of anodization on the hemisphere curves were further investigated. The branching and bending phenomena of nanopores reported here are shown to be deeply induced by electric fields rather than by the interpore distance mechanism [14].

## Experimental methods

The low-purity aluminum foil (99%, Alfa Aesar, Ward Hill, MA, USA) was used by our two-step HPA method. The plate was cut into a piece of 1.5 × 1.0 cm in size and then electropolished in a mixture of HClO_4 _and ethanol (1:4 in volumetric ratio) at 20 V for 30 s at RT. The two-step HPA is performed in a 0.5-M oxalic acid at RT. First of all, the applied hybrid pulse was constructed from a positive square wave followed by another negative square wave with the duty ratio of 1:1. The voltage of 40 V was applied on the positive pulse, while the negative voltage of -2 V was adopted for the negative pulse. The period of a hybrid pulse was 2 s (1 s:1 s). The formation of AAO was performed for 1 h by means of the potentiostat (5000, JIEHAN Technology Corporation, Taichung, Taiwan), and the three-electrode electrochemical cell with the platinum mesh acted as the counter electrode, the gold-coated silicon, as the working electrode, and Ag/AgCl, as the reference electrode. In order to further study the behavior of the anodization process, the real-time time-current curves were recorded. After the anodization, the specimens were immersed in 5 wt.% phosphoric acid at 50°C for 60 min in order to remove all porous alumina structures. The second anodization process was subsequently conducted by the same pulse condition as the first anodization. The morphology and pore characteristics of AAO films were examined using a high-resolution field emission scanning electron microscope [HR-FESEM] (JSM-7001, JEOL, Tokyo, Japan).

## Results and discussion

**Figure 1:**
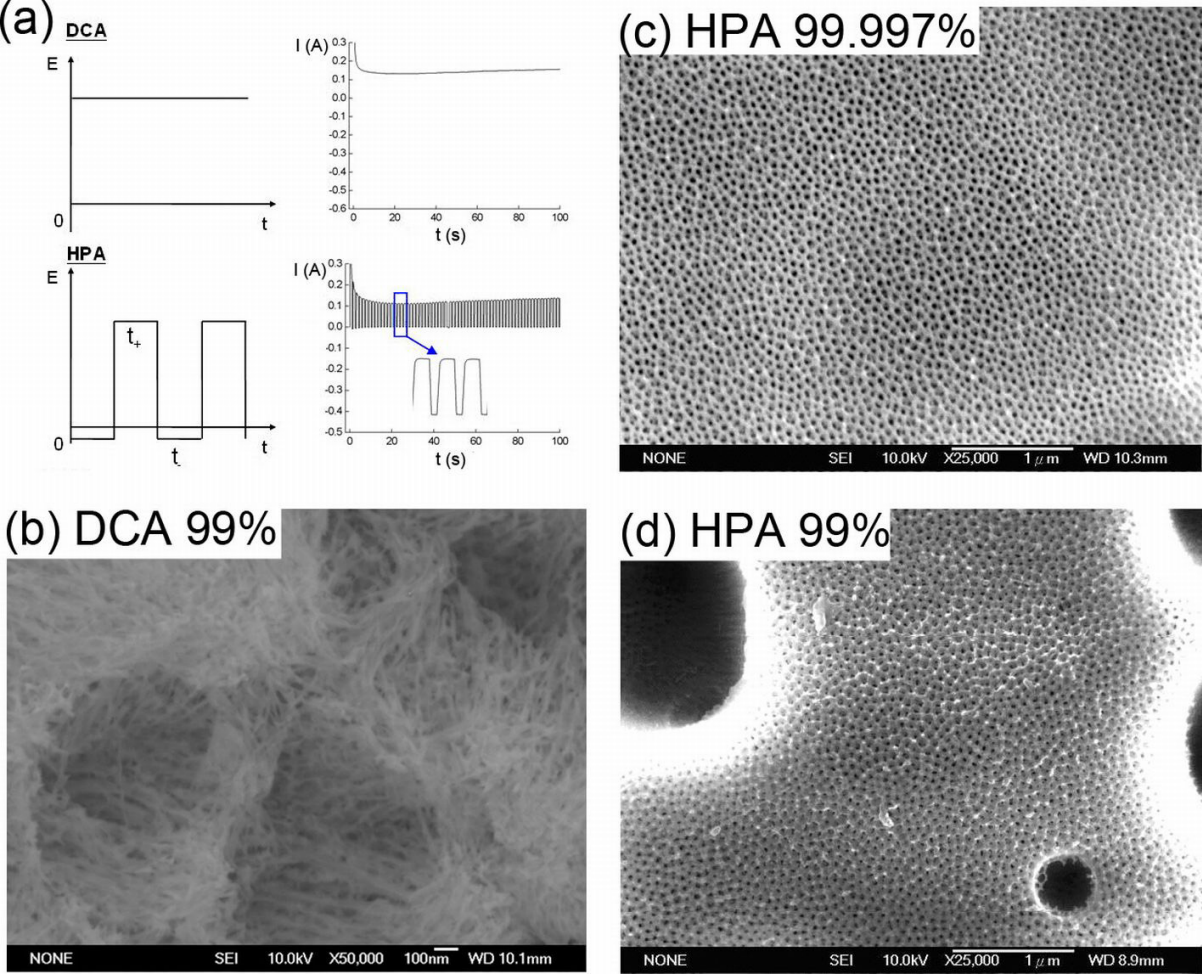
**Comparison of the relationship between the applied potential and corresponding *I*-*t *curve and HR-FESEM micrographs**. (**a**) Comparison of the relationship between the applied potential (*E*) and the corresponding current (*I*) as a function of time (*t*) using DCA and HPA. (**b**, **c**, **d**) HR-FESEM micrographs of a typical porous alumina surface morphology using DCA and HPA on the low-purity (99%) and high-purity (99.997%) Al foils.

**Figure 2:**
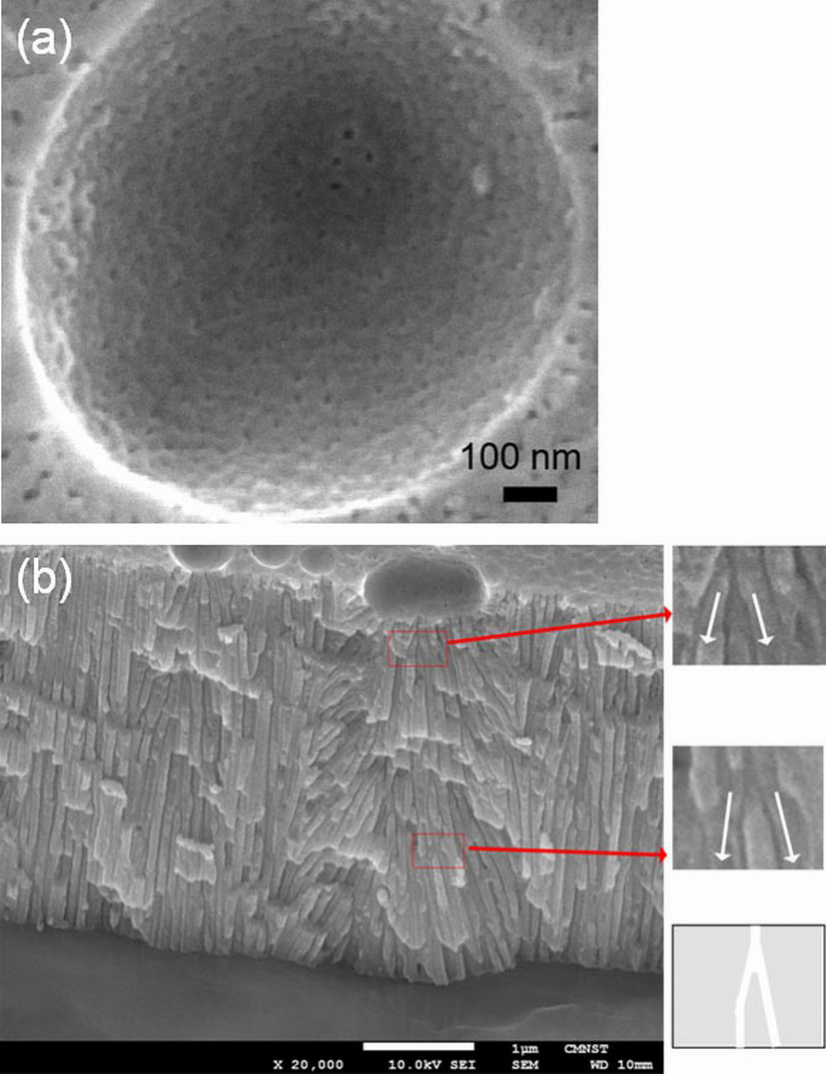
**HR-FESEM micrographs**. (**a**) Top view and (**b**) cross section of the magnified nanoporous alumina formed on the hemisphere curved surface.

Figure 1a shows the comparison of the applied potential and the corresponding *I*-*t *curve between DCA and HPA. The *I*-*t *curve during DCA is a continuous current while that in HPA is a square-wave current. Therefore, DCA leads to heat accumulation for the thermally enhanced dissolution effect, but HPA provides an effective liquid cooling at negative applied potential. Figures 1b,c,d show the HR-FESEM micrographs of a typical porous alumina surface morphology using DCA and HPA on the low-purity (99%) and high-purity (99.997%) Al foils. The AAO nanostructures from the low-purity Al foil were destroyed by DCA at RT due to a temperature-enhanced dissolution (Figure 1b). In the case of HPA, a smooth porous surface is obtained in the high-purity Al foil (Figure 1c) while several concavities of various sizes appeared on the low-purity Al surface (Figure 1d). Figures 2a,b show the HR-FESEM micrographs of the top view of the hemisphere curve and the cross section of the grown nanoporous alumina on the impurity-induced curved surface after HPA from the low-purity Al foil, respectively. Unlike the conventional flat Al surface, the growth of nanopores was found to develop along normal vectors of a hemisphere curve. Such a curved surface is generated by the impurity in the aluminum and the heat effect during the process, which is different from that of Yin et al. [14] by lithography and etching methods. It is noted that the formation of porous alumina which resulted from the impurity-induced hemisphere curved surface is deeply relying on the electrical fields rather than the interpore distance mechanism [14]. During the process of applying positive potentials to Al foil, which is seen as a conductor, the positive charge is repulsed to the surface. If the electrical field is not moving along the normal vector, it will force the positive charge to move until an equilibrium state is reached. Therefore, the directions of electrical field on a curved surface must be normal vectors at each position. In Figure 2b, with the magnified insets and schemes for the branched nanopore growth, the directions of pore channels in the concave surface were shown to be perpendicular to the curve. Other porous aluminum around the flat area except in the concave still remained to be in an original and normal straight shape. It is noted that the conventional pore channels in the concave area are not straight but bended towards the concave center. The bending phenomenon of the pore channel towards the central area and the alumina expanding force at the bottom of Al film have been reported by Yin et al. [14]. In our results, however, we find that there is no Al/Si interface formed in the anodization of Al foil, and bending directions are pointing towards the outer area of the concave. Such a unique feature is induced by electric fields with its generated curvature being larger than that reported by Yin et al. [14]. This mechanism of growth of nanopore structures is varied because these two curved surfaces are fabricated by different methods. Moreover, the inset picture shown in Figure 2b illustrates the Y-shaped branch in the pore channel positioned at the curve center. The channel branches were found again with the growth of porous alumina extending to several channels. Notice that the branch characteristic is not obvious outside the concave. It is different from the claim of Yin et al. [14] that the branch should occur due to the increased interpore distance in the curved area. According to the mathematical geometry of a hemisphere, the increased interpore distance *D*_t _can be estimated as follows:

(1) $$D_{t}=\left(r+d\right)\tan\left(\frac{\lambda }{2\pi r}\right),$$

where *r *is the radius of the concave (hemisphere), *d *is the thickness of the porous alumina, and *λ *is the original interpore distance in the curved surface. If the interpore distance is increased two times larger than that of the original interpore distance in the claim of Yin et al. [14], the branch will occur immediately. In our case, in Figure 2, the original interpore distance *λ *is 80 nm, *r *is 500 nm, and *d *is 3,000 nm. So the estimated increased interpore distance *D_t _*is about 89 nm. Thus, the branch behavior may not have resulted from increasing interpore distances, but from electric field variations.

**Figure 3:**
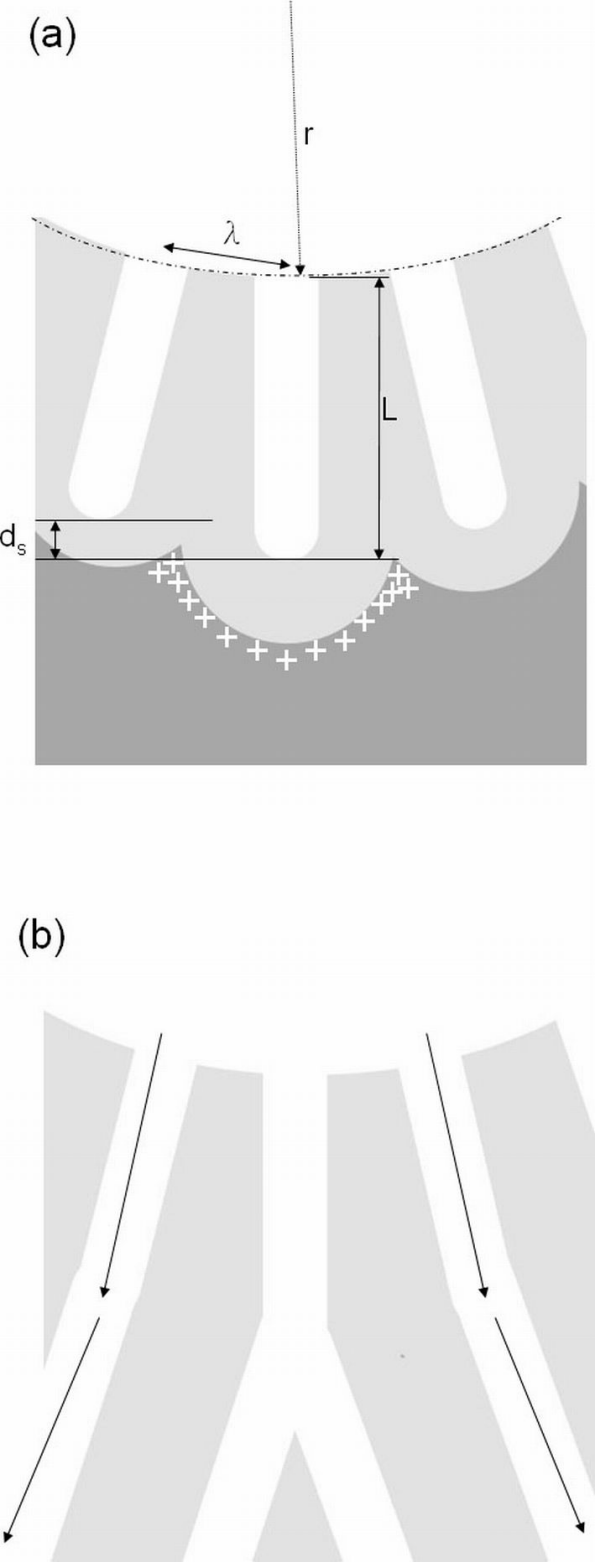
**Schematic diagrams of pore growth in the hemisphere curve**. (**a**) Electrical field distribution and depth difference and (**b**) bending phenomenon.

Figure 3a shows the schematic diagram of pore growth in the hemisphere curve. Owing to the different directions of pore growth, the depth differences of each pore channel are also depending on the growth time even if the growth rate of each pore is the same. The depth difference (*d*_s_) between the central and other pores can be estimated by the following formula:

(2) $$d_{s}=\left(r+L\right)\left(1-\cos\frac{\lambda }{2\pi r}\right),$$

where *L *is the length of the central pore channel and *r *and *λ *are the same as in Equation 1. Therefore, the depth difference also depends on the length of the pore channels. On the other hand, the electrical field strength on the interface of Al and alumina is concerned with the curvature of locations. The strongest electric field occurs in the cusp between pores. In the anodization of the flat Al foil, the depth for each pore channel is equal, so the influence of the strongest electric field is not clear. In the anodization of the curved Al, this unbalanced strongest electric field and small resistance from other pore walls lead to the central pore branching. With more branches being formed, the growth of the other pores will bend towards the outer area, as shown in Figure 3b. Therefore, a bending situation is more evident at the outer region of the concave center in the impurity-induced hemisphere curve.

**Figure 4:**
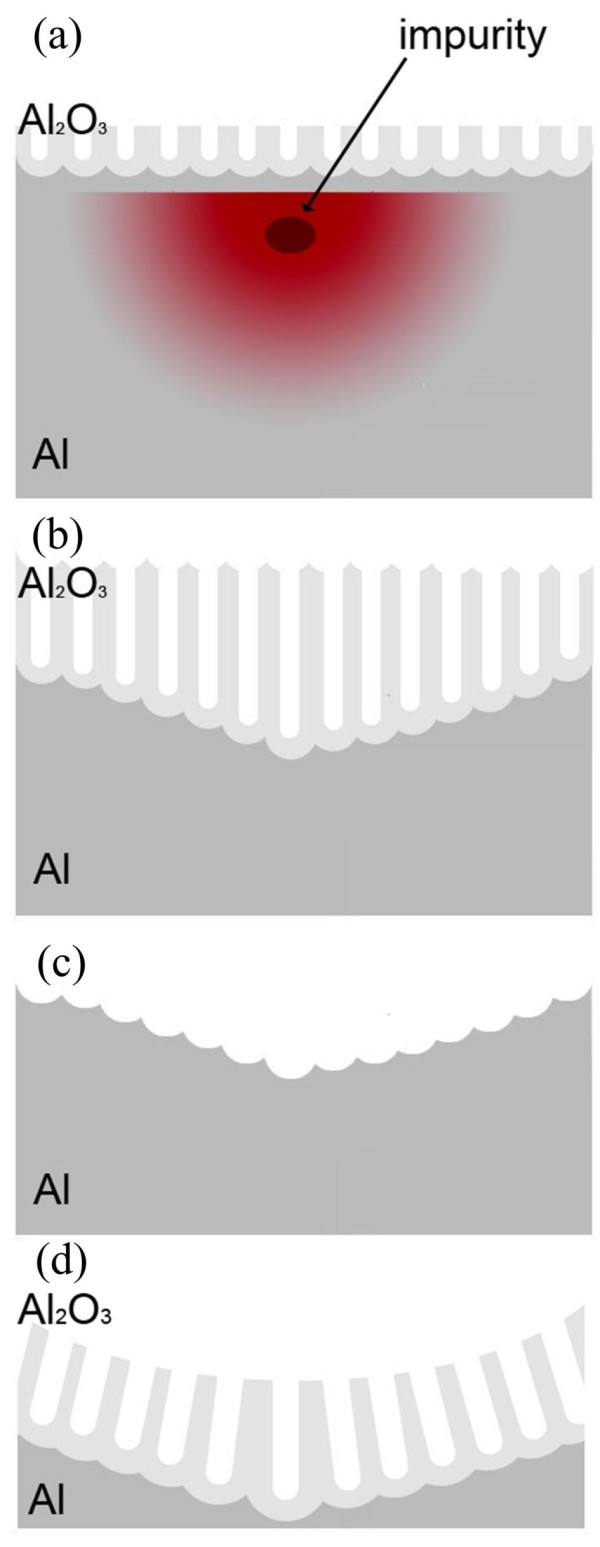
**Schematic procedure of hemisphere curved surface formation through the impurity during two-step anodization**. (**a**) Joule heat caused by impurity, (**b**) difference of grow rate, (**c**) removal of all porous alumina, and (**d**) the second anodization.

With regards to the impurity effect on the anodization of the low-purity Al, Figure 4 shows the schematic procedure of the hemisphere curved surface formation through the impurity during the HPA anodization method. The low-purity Al foil contains higher contents of impurities including the primary Si and Fe summed about 0.6% and others of Zn, Cu, Mg, Mn, and Ti elements being about 0.3% to 0.4%. When the anodizing process reaches these impurities, especially the elements with much higher electric resistivity, the local Joule heat significantly increases too. It is well known that both formation and dissolution of porous alumina are temperature-dependent processes, and the rate increases with increasing temperatures. It is noted that the resistivity of Si is much larger than that of Al (pure Al, 2.8 × 10^-8 ^Ω m and intrinsic Si, 3.2 × 10^3 ^Ω m at 20°C) in dominating and accelerating the electrochemistry reaction during the AAO process. The about eleven-order-higher resistivity Si possesses, the greater Joule heat the foil can generate around the spot of impurity. The HPA then took over the growth of nanostructures timely by suitably directing the growth of nanopores on the template. The impurity-induced thermal point source can be seen as a three-dimensional heat conduction problem. The heat flow in the *x*, *y*, and *z *directions can be expressed as follows:

(3) $$q_{\text{x}}=-kA_{\text{x}}\frac{\partial T}{\partial x};\;q_{\text{y}}=-kA_{\text{y}}\frac{\partial T}{\partial y};\;q_{\text{z}}=-kA_{\text{z}}\frac{\partial T}{\partial z}$$

where *q *is the heat transfer rate, *k *is thermal conductivity of Al, *A *is area, and $\frac{\partial T}{\partial x}$,$\frac{\partial T}{\partial y}$,and $\frac{\partial T}{\partial z}$ are the temperature gradients in *x*, *y*, and *z *directions, respectively. In this case, we can assume that the heat transfer rates are equal in three directions, so the heat-affected zone would be in a shape of a hemisphere due to the lower thermal conductivity of alumina, as shown in Figure 4a. The isothermal is along the surface of the hemisphere. Therefore, the formation and dissolution rate of porous alumina in the impurity-induced heat-affected zone are accelerated, as shown in Figure 4b. However, this difference is not observed in the porous alumina surface. It occurred only when all first-step porous aluminas are being removed completely. It is also seen that lots of hemisphere curves occur on the Al foil surface, as shown in Figure 4c. After the second anodization, porous aluminas are formed on the hemisphere curved surface along the normal vectors, as shown in Figure 4d.

Compared to the anodization of the flat Al foil, the porous alumina growth on the hemisphere curved surface could enlarge the whole surface area. From the viewpoint of geometry, the surface area is 2*πr*^2 ^in a hemisphere and *πr*^2 ^on a circle plane. That is, it is about two times the surface area by the anodization of Al foil compared with the hemisphere curved surface. It is very helpful for photocatalyst or sensor applications. By filling the nanopore with TiO_2 _materials like titanium dioxide [18], a large surface area can enhance photocatalyst performance. On the other hand, the particular branch structure of alumina pore channel can be used in fabricating Y-shaped carbon nanotubes [19].

## Conclusions

The behavior of porous alumina on a hemisphere curved surface has been demonstrated and examined by an HPA process on low-purity Al foil at RT. The hemisphere curve is formed through the Joule heat caused by the impurity and isotropic heat conduction phenomenon. The growth of nanopore is found to move along the normal vector of a hemisphere curve. The impurity with high electric resistivity can generate much more Joule heat around the impurity location for accelerating the electrochemistry response. Moreover, the pore channel positioned at the curve center had several branching due to different directions of each pore and the unbalanced strongest electrical field at the edge of the pore at the bottom. As branching is formed in the central channel, the other pore growth is bending towards the outer area, while the conventional research results claim that the pore structures have been bended towards the concave center in a different way. The feature of branching and bending of pore structures on the high-curvature hemisphere curve is induced by electric field rather than the large interpore distance in the conventional low-curvature cavity. Such a process for enhancing the AAO surface area is cost-saving for potential photocatalyst or sensor applications or Y-shaped carbon nanotube fabrications in the future.

## Competing interests

The authors declare that they have no competing interests.

## Authors' contributions

C-KC conceived the experiment of AAO formation using HPA compared to DCA, carried out the mechanism of branched AAO formation on the hemisphere curved surface with M-WL, and corrected and finalized the manuscript. M-WL carried out the experiment with H-CC, participated in the discussion of branched AAO formation mechanism, and drafted the manuscript. C-TL participated in the manuscript revision and in the mechanism discussion. H-CC carried out the experiment with M-WL and participated in the mechanism discussion. All authors read and approved the final manuscript.
